# Silent Threat: A Complex Presentation of Testicular Adrenal Rest Tumors in a Male With Congenital Adrenal Hyperplasia

**DOI:** 10.1002/ccr3.71498

**Published:** 2025-11-17

**Authors:** Shristi Gupta, Aron Neupane, Kshitiz Parajuli, Sagar Bhagat, Nischal Timsina, Raja Babu Gupta

**Affiliations:** ^1^ Birat Medical College Teaching Hospital Morang Nepal; ^2^ Department of Pediatrics Birat Medical College Teaching Hospital Morang Nepal

**Keywords:** adrenal rest tumors, congenital adrenal hyperplasia, hypertensive crisis, pediatrics, precocious puberty

## Abstract

Congenital adrenal hyperplasia (CAH) with testicular adrenal rest tumor (TART) is a sequela of 21‐hydroxylase deficiency in the background of adrenal hyperplasia in most cases. We report a case of a 10‐year‐old boy who presented with a 7‐day history of headache, vomiting, and abdominal pain, and had a blood pressure of 200/140 mmHg with a pulse rate of 84 bpm, consistent with a hypertensive emergency. Clinical presentation showed precocious puberty with virilization. The patient appeared like an adult male with well‐developed secondary sexual characters and Tanner staging IV was assessed. Hard nodular testicular swellings were present on both sides. Regular laboratory investigations showed elevated urea and creatinine, suggesting renal failure. Imaging revealed bilateral enlarged adrenal glands and right testes were found to have a homogeneous hypoechoic mass. Ophthalmoscopy confirmed Grade IV retinopathy. After comprehensive evaluation and investigations, the final diagnosis was made to be CAH with right‐sided TART with hypertensive emergency with renal AKI and grade IV hypertensive retinopathy. He was treated initially with antihypertensives followed by hydrocortisone and NSAIDs. Early diagnosis is significant in CAH with TART to prevent infertility and irreversible testicular damage.

**Summary**

- Testicular adrenal rest tumors are common in pediatric groups with congenital adrenal hyperplasia and lead to infertility if left untreated.
- Early detection through routine imaging can help prevent complications when appropriate glucocorticoid therapy is instituted, and complications like hypertension, retinopathy, and renal impairment can be prevented.

## 1 Introduction

Congenital adrenal hyperplasia (CAH) is a genetic disorder inherited in an autosomal recessive manner, characterized by a lack of enzymes necessary for glucocorticoid synthesis. The deficiency in glucocorticoids triggers an increase in the secretion of adrenocorticotropic hormone due to negative feedback, leading to adrenal gland enlargement.

Additionally, this condition results in the overstimulation of ectopic adrenal cell nests within the testes, which causes the formation of testicular adrenal rest tumors (TARTs). TARTs, sometimes referred to as testicular tumors associated with the adrenogenital syndrome, are believed to develop from misplaced adrenal cells that accompany the testes during embryonic development. The presence of testicular lesions in patients with CAH was initially reported by Wilkins and colleagues. The occurrence of TART is estimated to be up to 2 in 20,000 individuals, while the estimated prevalence of TART in males diagnosed with CAH is around 37%.

TART is benign but may cause infertility in males. Early diagnosis is important, and biannual Ultrasonography is advised in high‐risk groups [1].

We report a case of TART in a 10‐year‐old pediatric patient due to CAH complicated with hypertensive emergency with renal acute kidney injury with grade IV hypertensive retinopathy and its management.

## 2 Case Presentation

### 2.1 Case History/Examination

A 10‐year‐old boy presented to the emergency department of Birat Medical College Teaching Hospital with chief complaints of headache, vomiting, and pain in the abdomen for 4 days.

According to the parents, vomiting was 2–3 episodes per day, scanty in amount, and for the initial 4 days contained digested food particles, was non‐projectile, non‐bile‐stained and was associated with nausea.

There was pain in the periumbilical region, especially in the early morning, with no radiation and of mild severity.

The mother of the child had noticed the appearance of pubic hair since the age of 3 years and voice change since the boy turned 6 years old. There was no history of chronic headache, chronic vomiting, abnormal body movement, orifice bleed, decreased frequency of urination, loss of consciousness, earache, blurring of vision, scrotal swelling, and abdominal swelling. Fever and localized pain were absent. There was no prior history of hospital admission.

The child was born via SVD in a remote village health center. The parents did not remember the birth weight. There were no ANC visits. He was immunized according to the National Immunization Schedule.

The child is intellectually well but has low social performance and stammers while speaking. There is no family history of intractable headache and early signs of body development.

The patient first sought medical attention from a primary health care center. He developed symptoms such as restlessness, dizziness, anxiety and blurred vision and was rushed to the center. Here, the blood pressure was noted to be 220/120 mmHg. He was then referred to a tertiary care center where the BP was noted to be 230/120 mm of Hg. Inj. Labetalol 20 mg, Tab. Amlodipine 10 mg and Inj. Furosemide 20 mg were administered to control hypertension. The patient was then referred to our tertiary care center in view of PICU (Pediatric Intensive Care Unit) with a provisional diagnosis of precocious puberty with hypertensive emergency.

On general physical examination, BP was found to be 200/160 mm of Hg. The pulse was found to be 84 bpm, measured in the right radial artery with normal character and volume. All peripheral pulses were palpable. Temperature and oxygen saturation were under normal limits. There were no signs of increased JVP. PILCODE was nil. The GCS was 15/15.

The patient looked like an adult with a muscular appearance, mustache, broad hands and limbs, axillary hair and pubic hair, and an 8.5 cm penile length of Tanner staging IV. There was a presence of hard, non‐fluctuant, non‐tender testicular swelling over the right upper pole. Under anthropometry findings: weight: 40 kg, height: 1.43 m, body mass index (BMI): 19.5 kg/m^2^, upper segment (US): 0.79 m, lower segment (LS): 0.67 m, and arm span: 1.40 m were noted. The child had slightly longer upper limbs compared to lower limbs.

### 2.2 Differential Diagnosis, Investigations, and Treatment

Laboratory investigations showed that the child had low hemoglobin and RBC count. There was severe renal impairment (urea: 112 mg/dL and creatinine: 4.08 mg/dL), suggesting acute kidney injury. Serum Na was normal but K was low.

The patient presented with LH at 0.1 mIU/mL, FSH at 0.001 mIU/mL, subclinical hypothyroidism (T3 at 2.8 pg/mL, T4 at 1.3 ng/dL, TSH at 6.38 microIU/mL), AFP was normal at 2.4 ng/mL (normal range 10–20 ng/mL), high testosterone level (436 ng/dL), normal cortisol level (41.2 ng/mL) and high ACTH value: 303 pg/mL (normal range 6–48 pg/mL).

While in PICU hypertensive emergency was managed with Labetalol infusion.

**FIGURE 1:**
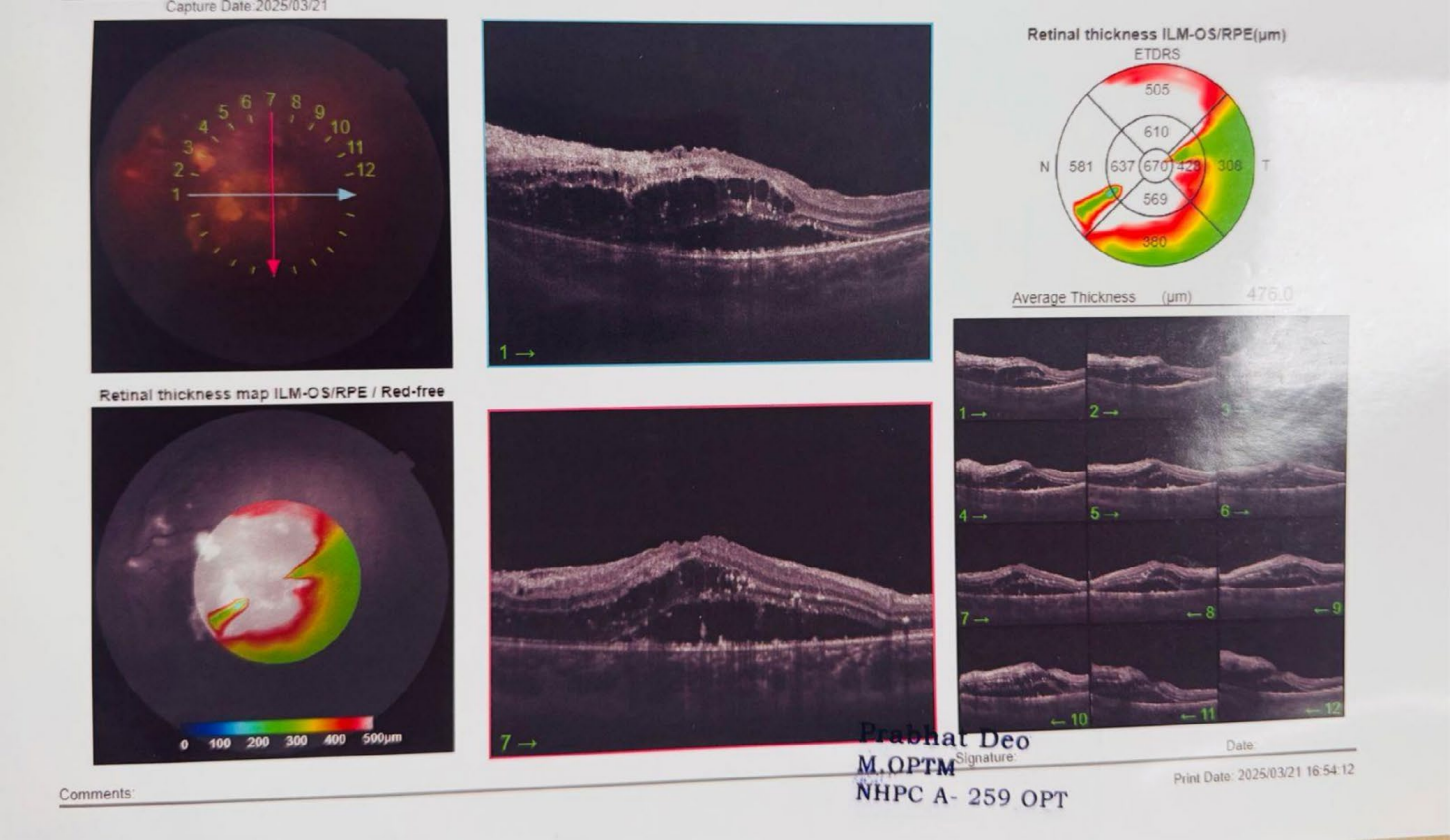
Grade 4 hypertensive retinopathy.

**FIGURE 2:**
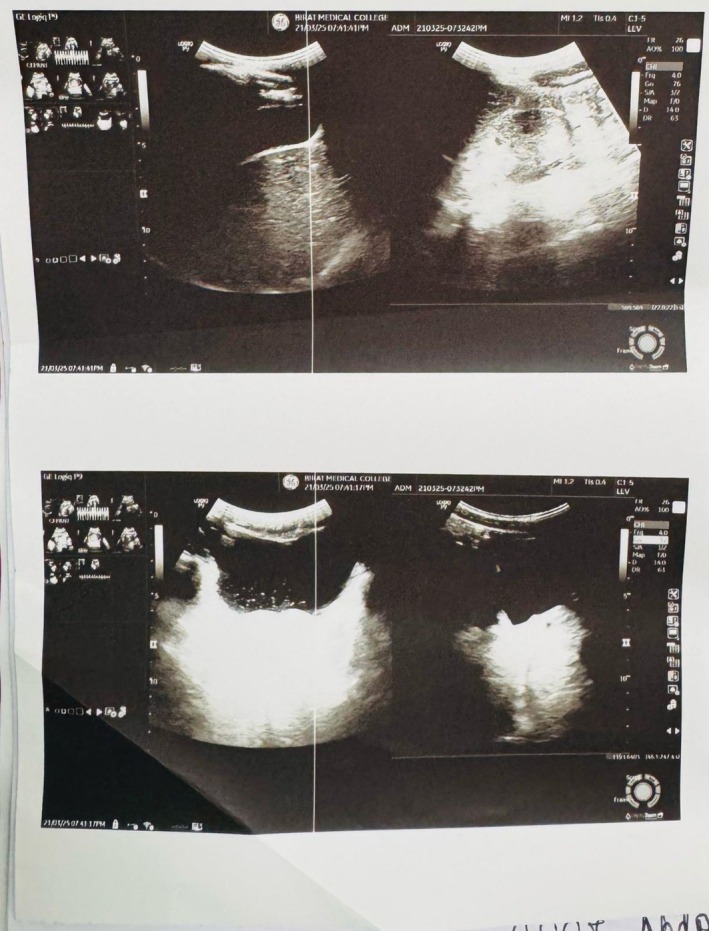
Bilateral enlarged adrenal glands.

Ophthalmology consultation was done which showed Grade IV retinopathy (Figure 1). Similarly, an endocrinology consultation was done and hydrocortisone was started in view of congenital adrenal hyperplasia. CT scan of the abdomen showed bilateral enlarged adrenal glands with maintained shape suggesting adrenal hyperplasia (Figure 2). Homogenous soft tissue density lesion was noted in the head region of the right testis, possibly arising from the head of the epididymis, measuring 15 × 12 mm with increased vascularity.

A few subcentimetric lymph nodes were noted in both inguinal regions, one measuring 13.3 × 6.3 mm on the left side.

During his PICU stay, the patient was treated with Amlodipine, Spironolactone, Hydrocortisone, and ketorolac + tromethamine eye drops.

Differentials including congenital adrenal hyperplasia with TART, Leydig cell tumor, and teratoma were considered, but excluded on the basis of biochemical reports and radiological imaging. The significantly high ACTH level (303 pg/mL) along with bilateral adrenal enlargement on the CT scan confirmed the diagnosis of congenital adrenal hyperplasia with an ACTH‐dependent testicular mass. The lesion was uniform, clearly defined, and situated at the head of the right epididymis—characteristics typical of TART. The hormonal analysis revealed elevated testosterone alongside low LH and FSH, with normal AFP and cortisol levels, suggesting adrenal tissue activity driven by ACTH instead of a primary testicular tumor. Leydig cell tumor was ruled out since these tumors are typically unilateral, not linked with CAH, show no ACTH increase, and do not respond to steroid treatment. Teratoma was excluded because imaging showed no heterogeneous, cystic, or calcified regions and AFP levels were normal. Accordingly, considering the biochemical indicators of CAH, imaging characteristics, and hormone profile, the definitive diagnosis of congenital adrenal hyperplasia with a right‐sided TART was confirmed.

After all the investigations, the final diagnosis of congenital adrenal hyperplasia with right‐sided TART with hypertensive emergency with renal AKI with Grade IV hypertensive retinopathy was made.

The patient was advised for further stay as BP was not in normal range but the patient's parents requested to get discharged and were discharged as per the demand.

Lab results on discharge showed urea at 108, serum creatinine at 3.44, serum sodium at 140, and serum potassium at 4.6.

Further ACTH stimulated 11‐Deoxycortisol test was planned to be sent but the patient party refused due to financial constraints. Any further genetic testing could not be accomplished as well.

## 3 Conclusion and Results

On discharge, the patient was advised to take Hydrocortisone 15 mg, Amlodipine 5 mg, Furosemide/Spironolactone 25/50 mg, Spironolactone 50 mg, ketorolac + tromethamine eye drops, monitor blood pressure twice a day, and follow up in the pediatrics OPD and ophthalmology OPD after 2 weeks.

During follow up, tests were again carried out. The blood pressure and potassium levels were in the normal range, the tumor size was reduced and the general condition of the patient improved. The patient's family refused to carry out any further tests due to financial restraints. On communicating with the patient on the telephone, he is doing well with blood pressure levels and has not had any hospital admissions ever since. However, he is in close contact with the hospital.

## 4 Discussion

Congenital adrenal hyperplasia (CAH) is a hereditary condition that impacts the adrenal gland's ability to produce steroids. It results from the deficiency of steroidogenesis enzymes, leading to a reduced production of cortisol and primarily aldosterone. As a result, the production of Adrenocorticotropic Hormone (ACTH) by the pituitary gland rises, causing hyperplasia of the adrenal glands [2].

The genetic alterations resulting in steroidogenesis defects are categorized as CAH and include the subsequent enzymes and proteins:

- 21‐hydroxylase (21‐OH)
- 11β‐hydroxylase (11β‐OH)
- 3β‐hydroxysteroid dehydrogenase isoform‐2 (3β‐HSD‐2)
- 17α‐hydroxylase/17,20‐lyase (17α‐OH)
- P450 oxidoreductase (POR)
- Steroidogenic acute regulatory protein (StAR)
- Cholesterol side chain cleavage enzyme (SCC) [3]

The most common deficiency among them is 21‐Hydroxylase which accounts for approximately 95% of the cases with a global incidence of 1 in 15,000 to 20,000 births.

Likewise, the global incidence of the 11β‐OH deficiency variant is estimated to range from 1 in 100,000 to 200,000 live births. 11β‐OH catalyzes the last stage of steroid synthesis and converts 11‐deoxycortisol to cortisol. These enzymes are essential for the synthesis of cortisol and aldosterone. In the most severe salt‐wasting version of the condition, there is a deficiency of both cortisol and aldosterone. However, excessive mineralocorticoid activity in CAH, like 11β‐OH deficiency, results in hypertension, hypokalemia and metabolic alkalosis due to the accumulation of aldosterone precursors [4].

Hypertension is caused by the accumulation of deoxycorticosterone resulting in water and sodium retention, increased plasma volume and eventually hypertension [5]. A lack of cortisol prompts feedback stimulation for ACTH release from the pituitary gland. Increased ACTH levels may lead to hyperplasia of the adrenal cortex, buildup of cortisol precursors, and rerouting of steroidogenic pathways, leading to the overproduction of androgens or precursors of mineralocorticoids [6]. The patient's presentation with extremely high blood pressure, along with Grave IV hypertensive retinopathy and Acute Kidney Injury, highlights a hypertensive emergency, which is the most severe manifestation in 11‐beta‐hydroxylase deficiency along with multiorgan damage. Due to the unavailability of specific biochemical tests, we assumed 11‐beta‐hydroxylase deficiency to be the etiology. Breil et al. suggest that CAH due to 11βOHD should be suspected in children with arterial hypertension, tall stature and precocious puberty [7, 8, 9].

CAH patients develop many complications which can be detected in early childhood. A significant and often observed complication in male patients with CAH is the occurrence of testicular tumors. These tumors were initially documented in 1940 by Wilkins and others. Since that time, numerous papers have detailed testicular tumors, primarily in the form of case reports. Due to their morphological and functional similarity to adrenal tissue, they are referred to as “testicular adrenal rest tumors” (TARTs) [2]. TARTs are benign testicular masses that arise from aberrant adrenal cells which descend with the testis during embryogenesis. One study found it occurs in the background of CAH in 37% of the patients while another found that TART occurred in 14%–86% of males with classic CAH, with an average prevalence of 25% in adolescents and 46% in adult men. The usual age of presentation of TART is 20–40 years [6, 10, 11].

We reported an earlier onset of TART in our case.

The incidence of TART is up to 2 in 20,000. TART was first defined by Wilkins in 1940. Despite various etiological theories, ACTH is acknowledged as the key factor that facilitates the formation of TART [6]. The patients are often asymptomatic but can lead to infertility and chronic testicular pain. TART less than 2 cm is difficult to identify through physical examination, making early imaging and distinguishing it from Leydig cell tumors crucial factors [12]. They are more prevalent after puberty [13] and are typically benign, bilateral, and multiple. Ultrasound can detect nodules as small as 2 mm, making annual testicular ultrasound essential for at‐risk individuals. MRI is considered unreliable for this purpose. Currently, no definitive treatment for TART exists; interventions mainly aim to restore fertility in adults. Although intensified glucocorticoid therapy is sometimes used, its efficacy is unproven, and it carries risks such as hypertension, striae, weight gain, and reduced final height [14]. We were able to identify TART in the form of a homogeneous soft tissue density lesion in the head region of the right testis with increased vascularity. Literature review showed TART is usually bilateral; however, we reported a unilateral, singular tumor.

Even in well‐controlled CAH cases, TART has been reported. Recent studies reveal that semen quality in men with CAH is significantly compromised, with all cases deemed pathological based on WHO criteria [15]. Due to the central location of the tumors near the mediastinum testis, the compression of seminiferous tubules can result in obstructive azoospermia and irreversible testicular damage [2].

In such a unique case of CAH with TART, glucocorticoid therapy is the choice of treatment in reversing all the irregularities and prompting recovery of affected patients. Management of hypertension using glucocorticoid therapy results from the feedback inhibition of the hypothalamus and the pituitary gland, leading to a decrease in ACTH release. As a result, the synthesis of deoxycorticosterone and androgens is decreased, reducing blood pressure and hindering additional masculinization [5]. Likewise, this escalation in glucocorticoids can result in a decrease in tumor size by inhibiting the same ACTH secretion, which in turn enhances testicular function [2].

The family decided not to seek further medical advice for their child because of financial limitations, the lack of access to tertiary healthcare facilities in the area where they live—in a rural part of Nepal—and the social stigma associated with this ailment. The child's prognosis remains guarded.

Early diagnosis helps to overcome challenges and ensures effective management of this complex condition. A multidisciplinary approach is necessary in managing such cases. The role of health insurance can not be overlooked.

## Author Contributions

**Shristi Gupta:** conceptualization, data curation, formal analysis, investigation. **Aron Neupane:** writing – original draft, writing – review and editing. **Kshitiz Parajuli:** writing – original draft, writing – review and editing. **Sagar Bhagat:** investigation, methodology, supervision, visualization. **Nischal Timsina:** investigation, methodology, supervision, visualization. **Raja Babu Gupta:** investigation, methodology, supervision, visualization.

## Consent

Written informed consent was obtained from the patient to publish this report in accordance with the journal's patient consent policy.

## Conflicts of Interest

The authors declare no conflicts of interest.

## Data Availability

The data underlying this case report are available from the corresponding author upon reasonable request.
